# Age-Associated Microbial Dysbiosis Promotes Intestinal Permeability, Systemic Inflammation, and Macrophage Dysfunction

**DOI:** 10.1016/j.chom.2018.03.006

**Published:** 2018-04-11

**Authors:** Netusha Thevaranjan, Alicja Puchta, Christian Schulz, Avee Naidoo, J.C. Szamosi, Chris P. Verschoor, Dessi Loukov, Louis P. Schenck, Jennifer Jury, Kevin P. Foley, Jonathan D. Schertzer, Maggie J. Larché, Donald J. Davidson, Elena F. Verdú, Michael G. Surette, Dawn M.E. Bowdish

## Main Text

(Cell Host & Microbe *21*, 455–466; April 12, 2017)

The authors would like to clarify that several experiments in the paper as detailed here were performed simultaneously. Specifically, the histology slides represented in Figures 1G, 2E, and 3H were analyzed by a blinded reviewer and quantified using the same pathology scale. The experiments yielding data on intestinal permeability (Figures 3D and 5B) and ELISA data (Figures 1E, 1F, 2B, 3G, and 3I) were performed simultaneously to minimize inter-experimental error. The relevant comparisons (e.g., age, genotype, SPF/germ-free) were presented in separate figures/panels to facilitate the narrative of the manuscript, but the statistical analysis was performed and presented based on analysis of the entire dataset. Additionally, the sentence “Mice were deprived of food 4 hr prior to and both food and water 4 hr following an oral gavage using 200 ml of 0.8 mg/ml FITC-dextran” should state “80 mg/ml FITC-dextran.” The authors apologize for this error, and it has been corrected online.

