# Supplementary material for: Age-Associated Microbial Dysbiosis Promotes Intestinal Permeability, Systemic Inflammation, and Macrophage Dysfunction
Source: Cell Host Microbe. 2017 Apr 12;21(4):455–466.e4. doi: 10.1016/j.chom.2017.03.002 (PMC5392495; doi:10.1016/j.chom.2017.03.002)
Supplement: Document S1. Figure S1 [file mmc1.pdf]

Cell Host & Microbe, Volume *21*

Supplemental Information

Age-Associated Microbial Dysbiosis Promotes Intestinal Permeability, Systemic Inflammation, and Macrophage Dysfunction

Netusha Thevaranjan, Alicja Puchta, Christian Schulz, Avee Naidoo, J.C. Szamosi, Chris P. Verschoor, Dessi Loukov, Louis P. Schenck, Jennifer Jury, Kevin P. Foley, Jonathan D. Schertzer, Maggie J. Larché, Donald J. Davidson, Elena F. Verdú, Michael G. Surette, and Dawn M.E. Bowdish

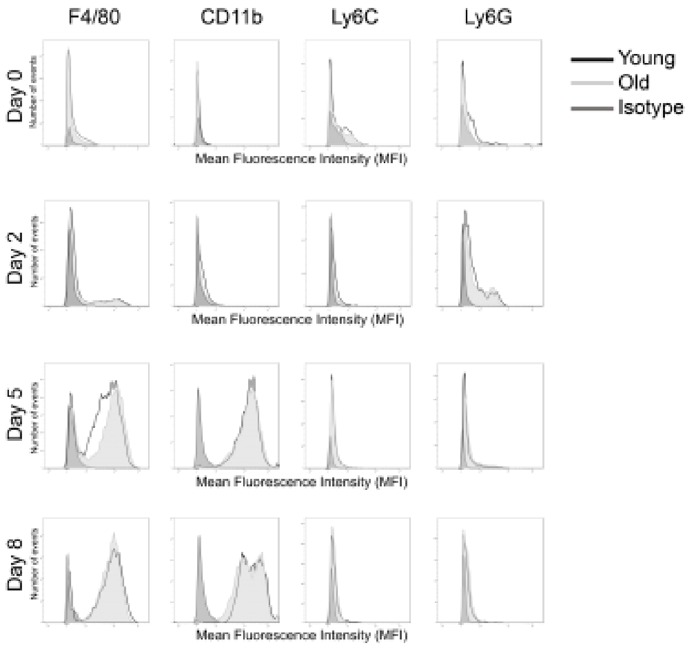

Figure S1 related to Figure1. Macrophage maturation does not differ with age. Histograms showing expression of key maturity markers (CD11b, F4/80, Ly6C and Ly6G) on differentiating bone marrow at days 0, 2, 5 and 8. Bone marrow was derived from young (3 mo and old (18-20 mo) mice and differentiated in M-CSF-enriched media. Bone marrow from one representative mouse of at least three is shown; cells were analyzed using flow cytometry. There were no statistically significant differences in the levels of expression of the markers tested at Day 8, when macrophages were used for experiments.
