# Supplementary material for: Age-Associated Microbial Dysbiosis Promotes Intestinal Permeability, Systemic Inflammation, and Macrophage Dysfunction
Source: Cell Host Microbe. 2017 Apr 12;21(4):455–466.e4. doi: 10.1016/j.chom.2017.03.002 (PMC5392495; doi:10.1016/j.chom.2017.03.002)
Supplement: Document S2. Article plus Supplemental Information [file mmc2.pdf]

Article

Cell Host & Microbe

Age-Associated Microbial Dysbiosis Promotes Intestinal Permeability, Systemic Inflammation, and Macrophage Dysfunction

Graphical Abstract

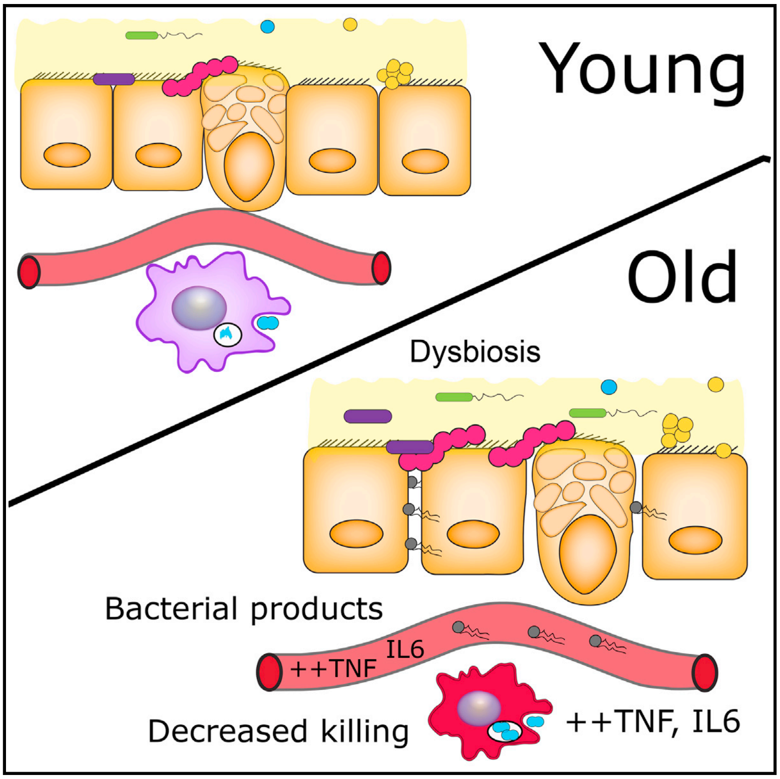

Highlights

- Age-associated inflammation drives macrophage dysfunction and tissue damage
- Mice under germ-free conditions are protected from age-associated inflammation
- Co-housing germ-free mice with old, but not young, mice increases age-related inflammation
- Age-related microbiota changes can be reversed by reducing TNF levels

Authors

Netusha Thevaranjan, Alicja Puchta, Christian Schulz, ..., Elena F. Verdú, Michael G. Surette, Dawn M.E. Bowdish

Correspondence

bowdish@mcmaster.ca

In Brief

Systemic inflammation increases with age, but the underlying causes are debated. Using young and old germ-free and conventional mice, Thevaranjan et al. demonstrate that age-related microbiota changes drive intestinal permeability, age-associated inflammation, and decreased macrophage function. Reducing TNF levels rescues microbiota changes and protects old mice from intestinal permeability.

Thevaranjan et al., 2017, Cell Host & Microbe *21*, 455–466
April 12, 2017 © 2017 The Authors. Published by Elsevier Inc.
http://dx.doi.org/10.1016/j.chom.2017.03.002

Cell Host & Microbe
Article

CellPress

Age-Associated Microbial Dysbiosis Promotes Intestinal Permeability, Systemic Inflammation, and Macrophage Dysfunction

Netusha Thevaranjan,[1,2,3,8] Alicja Puchta,[1,2,3,8] Christian Schulz,[1,2,3] Avee Naidoo,[1,2,3] J.C. Szamosi,[4] Chris P. Verschoor,[1,2,3] Dessi Loukov,[1,2,3] Louis P. Schenck,[3,4,5] Jennifer Jury,[4,6] Kevin P. Foley,[4,5] Jonathan D. Schertzer,[4,5] Maggie J. Larché,[6] Donald J. Davidson,[7] Elena F. Verdú,[4,6] Michael G. Surette,[3,4,6] and Dawn M.E. Bowdish[1,2,3,9,*]
[1]Department of Pathology and Molecular Medicine, McMaster University, Hamilton, ON L8N 3Z5, Canada
[2]McMaster Immunology Research Centre, McMaster University, Hamilton, ON L8N 3Z5, Canada
[3]Michael G. DeGroote Institute for Infectious Disease Research, McMaster University, Hamilton, ON L8N 3Z5, Canada
[4]Farncombe Family Digestive Health Research Institute, McMaster University, Hamilton, ON L8N 3Z5, Canada
[5]Department of Biochemistry & Biomedical Sciences, McMaster University, Hamilton, ON L8N 3Z5, Canada
[6]Department of Medicine, McMaster University, Hamilton, ON L8N 3Z5, Canada
[7]University of Edinburgh/MRC Centre for Inflammation Research, Queen's Medical Research Institute, Edinburgh EH16 4TJ, UK
[8]Co-first author
[9]Lead Contact
*Correspondence: bowdish@mcmaster.ca
http://dx.doi.org/10.1016/j.chom.2017.03.002

SUMMARY

Levels of inflammatory mediators in circulation are known to increase with age, but the underlying cause of this age-associated inflammation is debated. We find that, when maintained under germ-free conditions, mice do not display an age-related increase in circulating pro-inflammatory cytokine levels. A higher proportion of germ-free mice live to 600 days than their conventional counterparts, and macrophages derived from aged germ-free mice maintain anti-microbial activity. Co-housing germ-free mice with old, but not young, conventionally raised mice increases pro-inflammatory cytokines in the blood. In tumor necrosis factor (TNF)-deficient mice, which are protected from age-associated inflammation, age-related microbiota changes are not observed. Furthermore, age-associated microbiota changes can be reversed by reducing TNF using anti-TNF therapy. These data suggest that aging-associated microbiota promote inflammation and that reversing these age-related microbiota changes represents a potential strategy for reducing age-associated inflammation and the accompanying morbidity.

INTRODUCTION

In 1907, Elie Metchnikoff proposed that tissue destruction and senescence were consequences of chronic systemic inflammation, which occurred as a result of increased permeability in the colon and the escape of bacteria and their products (Metchnikoff, 1907). He believed that these bacterial products activated phagocytes and that the resulting inflammatory response caused deterioration of surrounding tissues. Indeed, aging is characterized by a state of chronic, low-grade, systemic inflammation (Franceschi et al., 2000). Higher than average levels of age-associated inflammation are a strong predictor of overall ill health, development of chronic inflammatory conditions, and all-cause mortality in the elderly. Although age-associated inflammation influences the aging process, it is unclear why levels of cytokines in the tissues and circulation increase with age. It has been theorized that gradual, cumulative, sub-clinical tissue damage occurs, which increases the burden of tissue repair and results in increasing background levels of pro-inflammatory cytokine production (Franceschi et al., 2000); however, the experimental evidence that would definitively prove this hypothesis is lacking.

In contrast, studies in *Drosophila* demonstrate that age-related changes in the microbiota increase intestinal permeability (Clark et al., 2015) and drive inflammation and mortality (Rera et al., 2012). Although it has been demonstrated that the microbial composition of the gut correlates with levels of circulating cytokines in the nursing home elderly (Claesson et al., 2012) and in old mice (Conley et al., 2016), it is not known whether this association is correlative or whether the gut microbiota are a driver of age-associated inflammation. If the latter is true, it would indicate that these age-related changes in composition are a form of microbial dysbiosis.

Herein we report that intestinal permeability increases with age in mice due to age-related microbial dysbiosis. We demonstrate that microbial products enter the bloodstream of aged mice where they trigger systemic inflammation (i.e., elevated levels of serum interleukin 6 [IL6]). Chronic exposure to inflammation alters macrophage function, rendering these cells poor killers of bacteria but potent producers of inflammatory cytokines, which ultimately contributes to the inflammatory state of the aged host. Using old (18–22 months)

© 2017 The Authors. Published by Elsevier Inc.
This is an open access article under the CC BY license (http://creativecommons.org/licenses/by/4.0/).
CrossMark

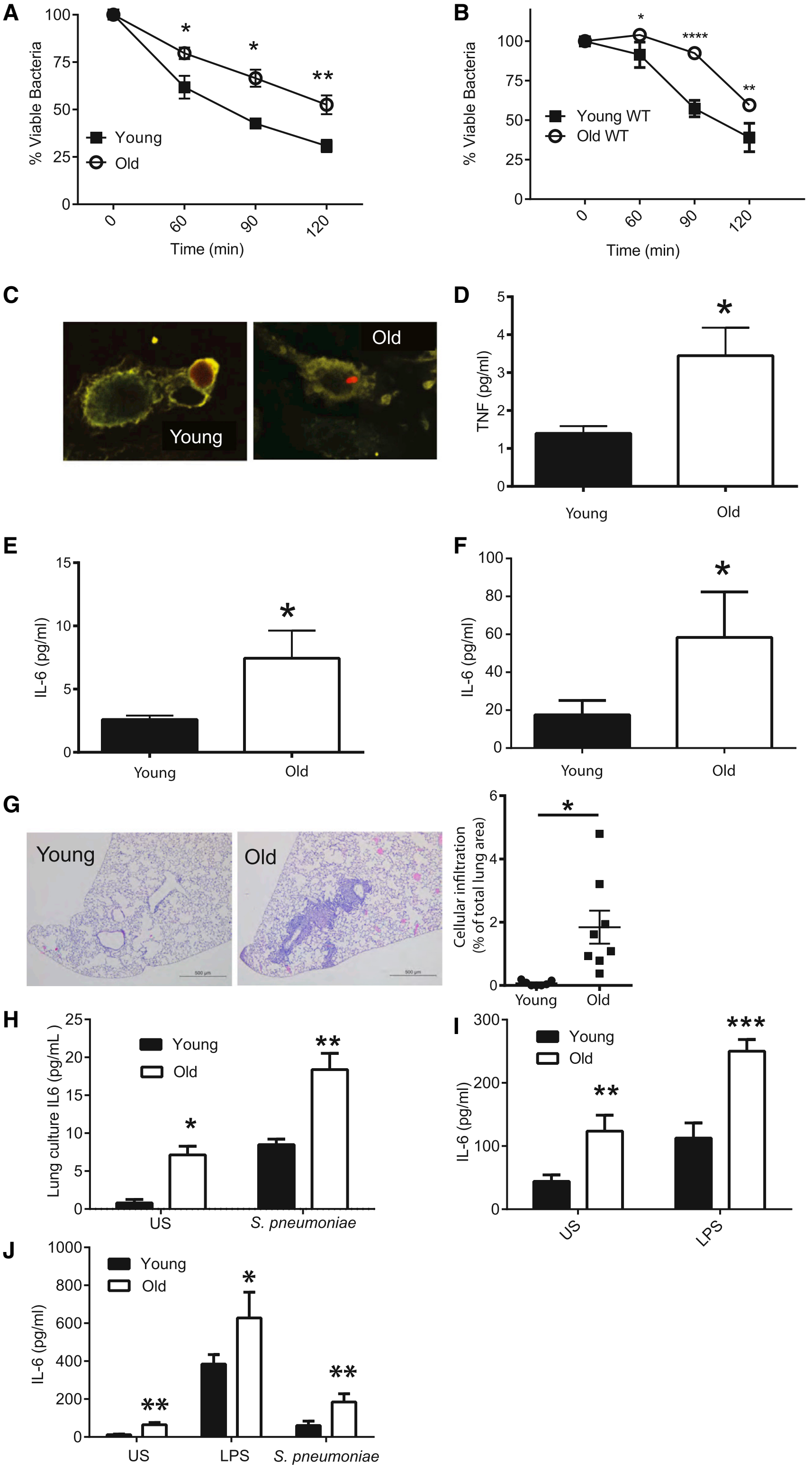

Figure 1. Inflammatory Responses Increase with Age

(A) Killing of *S. pneumoniae* by resident peritoneal macrophages isolated from young and old mice (n = 5).

(B) Killing of *S. pneumoniae* by bone marrow-derived macrophages from young and old C57BL/6 mice (n = 6).

(C) Intact tetramethylrhodamine (TRITC)-labeled *S. pneumoniae* was observed in macrophages derived from old mice, but not young mice, up to 4 hr post-infection.

(D–F) Levels of TNF (D) and IL6 (E) were higher in the plasma of old mice as was IL6 in slices of whole-lung homogenates from old mice (F).

(G) H&E stain of formalin-fixed histological sections from the lungs of young and old WT mice at 5× magnification. One representative image is at least five biological replicates. The degree of cellular infiltration within each image was measured by expressing the total area of the cellular infiltrate within the lung as a percentage of the total lung area.

(H) Lung slices were processed from the lungs of young and old mice and cultured in media. IL6 production was subsequently measured in the supernatant at 4 hr following stimulation with heat-killed *S. pneumoniae* or PBS control (n = 3, representative of two independent experiments).

(I) IL6 production in the whole blood of young and old WT mice following stimulation with LPS or a vehicle control (PBS) (n = 5–9).

(J) IL6 production from macrophages derived from young and old mice following 24-hr stimulation with a vehicle control (PBS), LPS, or *S. pneumoniae* as measured by ELISA (n = 6). Results represent mean ± SEM. Statistical significance was determined using the Mann-Whitney test or two-way ANOVA with Fisher's post-test where appropriate (*$p < 0.05$, **$p < 0.005$, and ***$p < 0.0005$).

germ-free mice, which do not have age-associated inflammation, we demonstrate that colonization with the microbiota from old mice drives the inflammation that accompanies aging.

RESULTS

TNF Drives Age-Associated Defects in Macrophage Function

We found that, after normalizing for differences in bacterial uptake between mice, resident peritoneal (Figure 1A) and bone marrow-derived (Figure 1B) macrophages from old wild-type (WT) mice (18–22 months) were impaired in their ability to kill *Streptococcus pneumoniae* as compared to those from young WT mice (10–14 weeks). Following internalization, bacterial lysis was observable in macrophages from young mice but reduced or delayed in old mice (Figure 1C). Maturation markers on macrophages from young and old mice were expressed at equal levels, indicating that differences observed with age were not due to altered differentiation or maturity (Figure S1, related to Figure 1).

Levels of pro-inflammatory cytokines, such as IL6 and tumor necrosis factor (TNF), in the circulation and tissues increase

CellPress

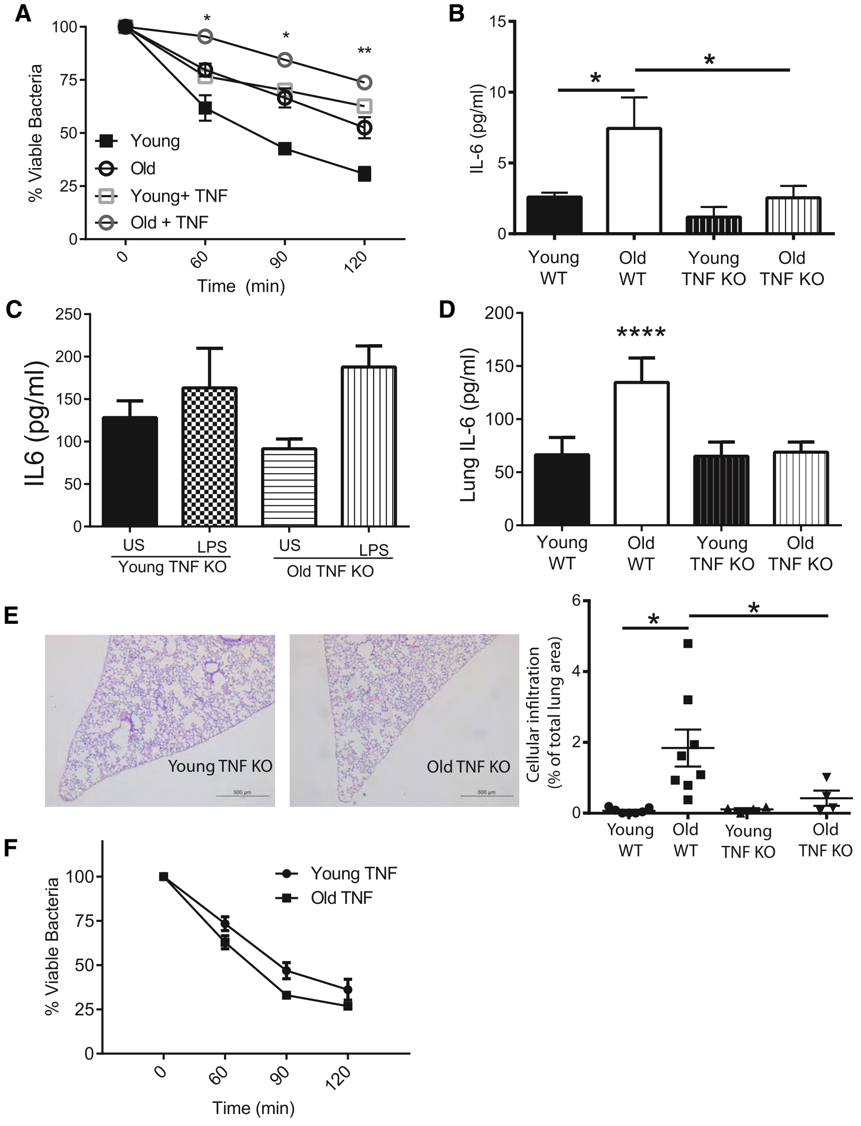

Figure 2. Chronic Exposure to TNF Contributes to Increased Inflammatory Responses and Tissue Damage that Occur with Age

(A) Young and old murine bone marrow macrophage-mediated killing of *S. pneumoniae* is decreased in the presence of 10 ng/mL exogenous TNF (n = 5).

(B) Unlike old WT mice, old TNF KO mice do not have increased levels of plasma IL6 (n = 3–10 mice per group, one of two independent experiments shown).

(C) IL6 production in the whole blood of young and old TNF KO mice following stimulation with LPS or a vehicle control (PBS) demonstrates that old TNF KO mice do not have higher inflammatory responses to LPS compared to young mice (n = 5).

(D) IL6 levels as detected by ELISA in whole-lung tissue homogenates were no higher in old TNF KO mice than in young TNF mice (n = 3).

(E) H&E stain of formalin-fixed histological sections of lungs of young and old TNF KO mice (20× magnification, one representative of at least four). The degree of cellular infiltration within each image was measured by expressing the total area of the cellular infiltrate within the lung as a percentage of the total lung area.

(F) Bone marrow-derived macrophages from young and old TNF KO mice do not differ in their ability to kill *S. pneumoniae* (n = 5). Results represent pooled data and are shown as mean ± SEM. Statistical significance was determined using the Mann-Whitney test or two-way ANOVA with Fisher's post-test where appropriate (*p < 0.05, **p < 0.005, and ***p < 0.0005).

with age, both in humans and mice (Bouchlaka et al., 2013; Franceschi et al., 2007). In keeping with previous reports, levels of TNF and IL6 in the circulation (Figures 1D and 1E) and IL6 in the lungs (Figure 1F) were higher in old mice. Peribronchiolar cellular infiltration was observed in the lungs of old mice in the absence of stimulation or overt infection (Figure 1G). Consistent with what others have shown, whole blood (Figure 1H) from old mice produced higher baseline levels of IL6 than that from young mice, and it produced more IL6 when stimulated with *S. pneumoniae* or lipopolysaccharide (LPS), demonstrating significantly enhanced pro-inflammatory responses to live bacteria and bacterial products. This phenotype was also observed using bone marrow-derived macrophages from old mice, which produced more IL6 following stimulation with LPS or *S. pneumoniae* compared to young mice (Figure 1I).

It has been frequently observed that individuals with higher levels of age-associated inflammation are at increased risk of both developing and dying from *S. pneumoniae* infection (Antunes et al., 2002; Yende et al., 2005). Furthermore, infusion of TNF into mice impairs anti-pneumococcal immunity and increases levels of *S. pneumoniae* in experimental models (Hinojosa et al., 2009). We therefore hypothesized that the chronically elevated levels of TNF that occur with age could have a direct effect on macrophage-mediated killing of *S. pneumoniae*. The exogenous addition of TNF (10 ng/mL) to culture media reduced bacterial killing by macrophages from young or old mice (Figure 2A), indicating that the decreased ability of old macrophages to kill *S. pneumoniae* could be due to higher levels of TNF.

Since acute exposure to TNF impaired macrophage killing of *S. pneumoniae*, we postulated that chronic age-associated inflammation, characterized by high systemic levels of TNF, might underpin the reduction in macrophage anti-bacterial activity. In contrast to WT animals, aged TNF knockout (KO) mice did not have greater levels of IL6 in the circulation in the steady state (Figure 2B), and when LPS was added to whole blood, old TNF KO mice did not produce higher amounts of IL6 than the stimulated blood of young mice (Figure 2C). Constitutive IL6 production was not affected by age in TNF KO lung slices (Figure 2D), and pulmonary cellular infiltrates were not observed in old mice, demonstrating protection from inflammation in the lungs (Figure 2E) Finally, bone marrow-derived macrophages from old TNF KO mice did not have impaired pneumococcal killing

in contrast to old WT mice (Figure 2F). Thus, age-associated inflammation and, more specifically, chronic exposure to TNF contribute to changes in macrophage function, resulting in decreased *S. pneumoniae* killing capacity.

Intestinal Permeability and Levels of Circulating Bacterial Products Increase with Age

Although our data demonstrated that the presence of TNF promoted systemic inflammation and impaired macrophage function, the cause of increased TNF production with age was unclear. Based on Metchnikoff's hypothesis that bacterial components from the gut microbiota could cause systemic inflammation, we investigated whether increased intestinal permeability and translocation of bacterial products occurred in aged mice.

Intestinal permeability was measured in WT mice (3, 12, 15, and 18 months old), by performing oral gavages with 3–5 kDa fluorescein isothiocyanate (FITC)-labeled dextran and measuring translocation of fluorescence into the plasma. Intestinal permeability increased with age (Figure 3A). We next assessed whether this was due to altered paracellular and/or passive permeability in the ileum and colon of young and old WT mice. Although there were no gross differences in intestinal architecture (Figure 3B), paracellular permeability was higher in the colons of old mice (Figure 3C), as determined by mucosal-to-serosal flux using ^{51}chromium-EDTA (^{51}Cr-EDTA). Consistent with evidence of increased permeability in the colon, where bacterial numbers are highest, levels of the bacterial cell wall component muramyl dipeptide (MDP) were also significantly higher in the plasma of old WT mice compared to young mice (Figure 3D). Thus, increased leakiness of the gut is a consequence of aging.

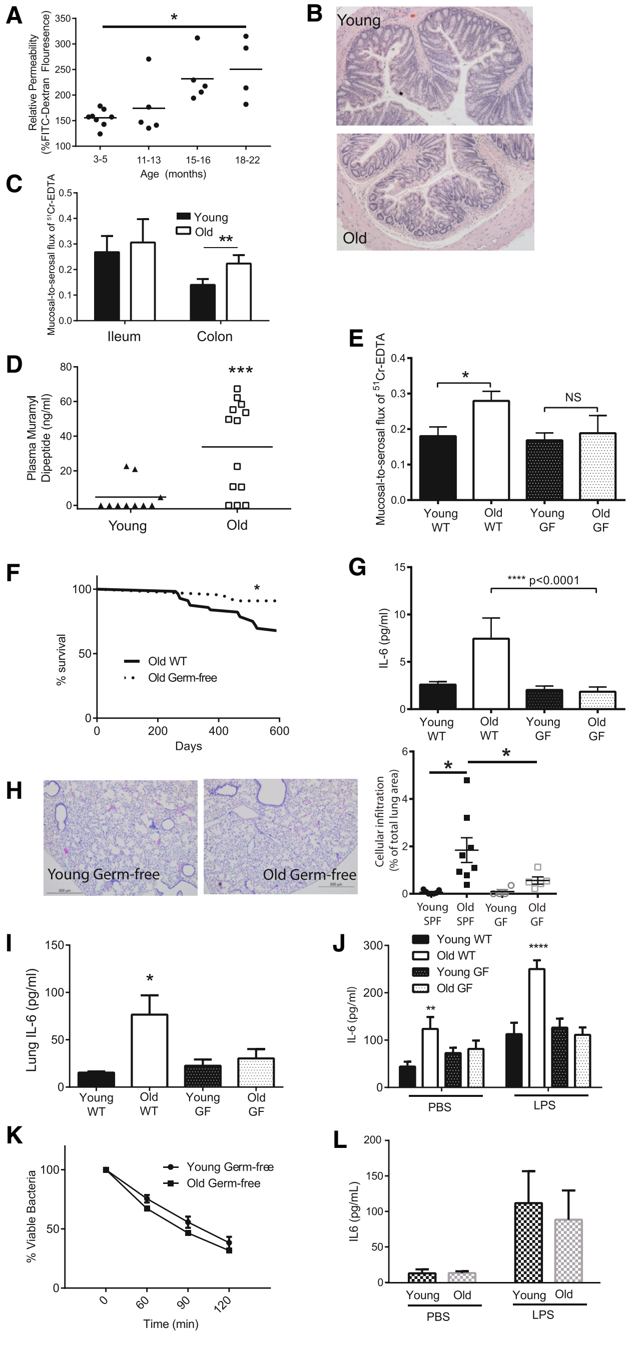

Figure 3. The Microbiota Increase Intestinal Permeability, Age-Associated Inflammation, Macrophage Function, and Longevity

(A) Intestinal permeability of aging mice (3, 12, 15, and 18 months old) was measured by FITC-dextran translocation to the circulation following oral gavage (n = 4–8), and it was found to increase significantly with age ($p < 0.007$, one-way ANOVA).

(B) Colons of young and old mice do not have detectable changes in either epithelial architecture or inflammatory infiltrate when measured as described in the STAR Methods.

(C) Mucosal-to-serosal flux of ^{51}Cr-EDTA as measured in Ussing chambers was used to measure the paracellular permeability of ileums and colons from young and old WT mice (n = 6–12).

(D) Circulating muramyl dipeptide (MDP) in the plasma of young and old WT mice as measured by nucleotide-binding oligomerization domain containing protein (NOD)-nuclear factor κB (NF-κB) promoter bioassay. Significant changes shown are relative to young WT mice.

(E) Mucosal-to-serosal flux of ^{51}Cr-EDTA as measured in Ussing chambers was used to measure the paracellular permeability of the colons from young and old GF mice (n = 5–8). There was no significant increase in permeability in old GF mice.

(F) Survival analysis showing all-cause mortality of WT and GF mice up to 600 days of life. Differences in the survival curves were analyzed by log rank (Mantel-Cox) test.

(G) Plasma cytokines are not higher than young GF mice and are lower than WT SPF mice (n = 3–5).

(H) Histological analysis of lung sections stained with H&E from young and old GF mice does not indicate any increased leukocyte infiltration with age (20× magnification; one representative image of at least five mice).

(I) IL6 levels in the lung homogenates of old GF mice are not higher than in young WT or young GF mice.

(J) IL6 production in the whole blood of young and old WT SPF and GF mice following stimulation with LPS or a vehicle control (PBS). Old GF mice do not have higher levels than young WT SPF or young GF mice (n = 5–9). Significant changes shown are relative to young WT mice.

(K) Macrophages from young and old GF mice do not differ in their ability to kill *S. pneumoniae* (n = 3). Results represent the mean ± SEM of three biological replicates.

(L) Bone marrow-derived macrophages from old GF mice do no not have decreased killing or produce more IL6 following stimulation with LPS or a vehicle control (PBS) in macrophages from young GF mice (n = 5). Statistical significance was determined using the Mann-Whitney test or two-way ANOVA with Fisher's post-test where appropriate (*$p < 0.05$, **$p < 0.005$, and ***$p < 0.0005$).

Germ-free Mice Are Protected from Age-Associated Inflammation and Dysregulated Macrophage Function

If the increase in circulating microbial products is a driving force in age-associated inflammation and mortality, we reasoned that germ-free (GF) mice, which have no detectable MDP in the circulation nor increased intestinal permeability with age (Figure 3E), would be protected. The proportion of GF mice that lived to 600 days was higher than the specific pathogen free (SPF) mice (Figure 3F). These GF mice were protected from age-associated inflammation, lacking the high circulating IL6 levels found in old control animals (Figure 3G). They also did not have peribronchiolar cellular infiltrates (Figure 3H) or increased levels of IL6 in the lungs (Figure 3I) compared to young GF mice. Furthermore, baseline and LPS-induced IL6 in whole blood did not increase with age in GF mice, in contrast to the significantly higher levels in old SPF/WT mice (Figure 3J). Finally, bone marrow-derived macrophages from old GF mice did not have impaired *S. pneumoniae* killing capacity (Figure 3K) or produce more IL6 than young GF mice either basally or after stimulation with LPS ex vivo (Figure 3L). These data demonstrate that chronic age-associated inflammation requires the presence of microbiota.

The Composition of the Microbial Community Influences Intestinal Permeability and Age-Associated Inflammation

We envisioned two possibilities that could explain how the microbiota drive age-associated inflammation. In the first, the presence of any microbiota, even minimal microbiota, could result in increased intestinal permeability. The second hypothesis was that microbial dysbiosis occurs with age to drive increased intestinal permeability.

To test these hypotheses, mice with a minimal microbiome were used. Mice were colonized with the altered Schaedler flora (ASF) (Dewhirst et al., 1999) on the C57BL/6 background and bred for two generations, during which time their microbiota diversified naturally as previously described (Slack et al., 2009). The result is a low-diversity microbial community. Similar to old SPF WT mice, old ASF-derived mice had greater intestinal permeability (Figure 4A), higher levels of plasma IL6 (Figure 4B), and higher IL6 production in whole blood following PBS or LPS stimulation (Figure 4C) than did young mice. Despite having minimal microbiota these mice also experienced age-related microbial dysbiosis (Figure 4D).

Although our data demonstrated that colonization with microbiota of initially limited diversity was sufficient to elicit age-associated changes in permeability and inflammation, we next investigated whether the microbial composition changes with age to determine if microbial dysbiosis might contribute to these phenotypes. Similar to what others have reported, we found that there were changes in both community structure (Figure 4E) and specific operational taxonomic units (OTUs) in the SPF mice between young and old mice (Table 1). To determine whether this dysbiosis could increase age-associated inflammation, young and old GF mice were colonized, via co-housing, with microbiota from either young or old SPF mice. The microbial dysbiosis that was evident in the fecal microbiota of the donor mice was maintained in the colonized recipient mice over the time course of this study (Figure 4E). After a minimum of 6 weeks, changes in paracellular permeability were assessed in the colonized mice. The paracellular permeability was measured from all the GF mice colonized with the microbiota sourced from old mice (n = 23 total, n = 11 young mice and n = 12 old mice) compared to young microbiota (n = 13, n = 6 young mice and n = 7 old mice). Microbiota sourced from old mice significantly increased paracellular permeability (Figure 4F). The age of the host did, however, influence the development of paracellular permeability. Young mice colonized with the microbiota from old mice had higher paracellular permeability, demonstrating that the composition of the microbiota can increase permeability. Although old mice colonized with the old microbiota had the greatest degree of permeability, old mice colonized with the young microbiota also had an increase in permeability, implying that there are age-related changes in the gut that predispose to barrier dysfunction (Figure 4G).

In addition to the paracellular permeability, the age of the host contributed to levels of circulating TNF (Figure 4H). Circulating TNF was measured from all the young GF mice (n = 13 total, n = 5 colonized with young microbiota and n = 8 colonized with old microbiota) and old GF mice (n = 11 total, n = 5 colonized with the young microbiota and n = 6 colonized with the old microbiota). Old recipients had higher levels of circulating TNF than young recipients (Figure 4H). Young GF mice that were colonized with old SPF microbiota had higher levels of plasma TNF than those recolonized with young SPF microbiota (Figure 4H), indicating that the specific composition of the aging microbial community contributes to age-associated inflammation but there are age-related changes that predispose old mice to increased inflammation when they are exposed to any microbiota (Figure 4I). These data indicate that the composition of the aged microbiota altered intestinal permeability but that the composition of the microbiota interacts with other age-related changes in the host to enhance systemic inflammation.

A Reciprocal Relationship between Age-Associated Inflammation and Microbial Dysbiosis

Our data demonstrate that the gut microbiota and/or age-related microbial dysbiosis can lead to increased gut permeability with age and result in age-associated inflammation. However, since expression of TNF has been shown to increase intestinal permeability in vitro (Söderholm et al., 2004) and anti-TNF treatment can alter intestinal permeability in vivo (Noth et al., 2012), we also considered the possibility that age-associated increases in TNF could exacerbate intestinal permeability and subsequent release of bacterial products. We hypothesized that, if age-associated increases in TNF promoted increased intestinal permeability, old TNF KO mice would be protected and would not have higher levels of circulating bacterial components than young TNF KO mice. Consistent with this, intestinal barrier function in old TNF KO mice was equivalent to young TNF KO mice, young SPF WT mice, and young or old GF mice (Figure 5A), and circulating levels of MDP in these mice did not increase with age (Figure 5B).

Although TNF is proposed to alter permeability of epithelial barriers, the mechanism remains unclear. We hypothesized that TNF may be a driver of microbial dysbiosis and that this might be an indirect way in which barrier function is decreased. If this hypothesis is correct then age-related microbial dysbiosis would be less pronounced in old TNF KO mice. To determine

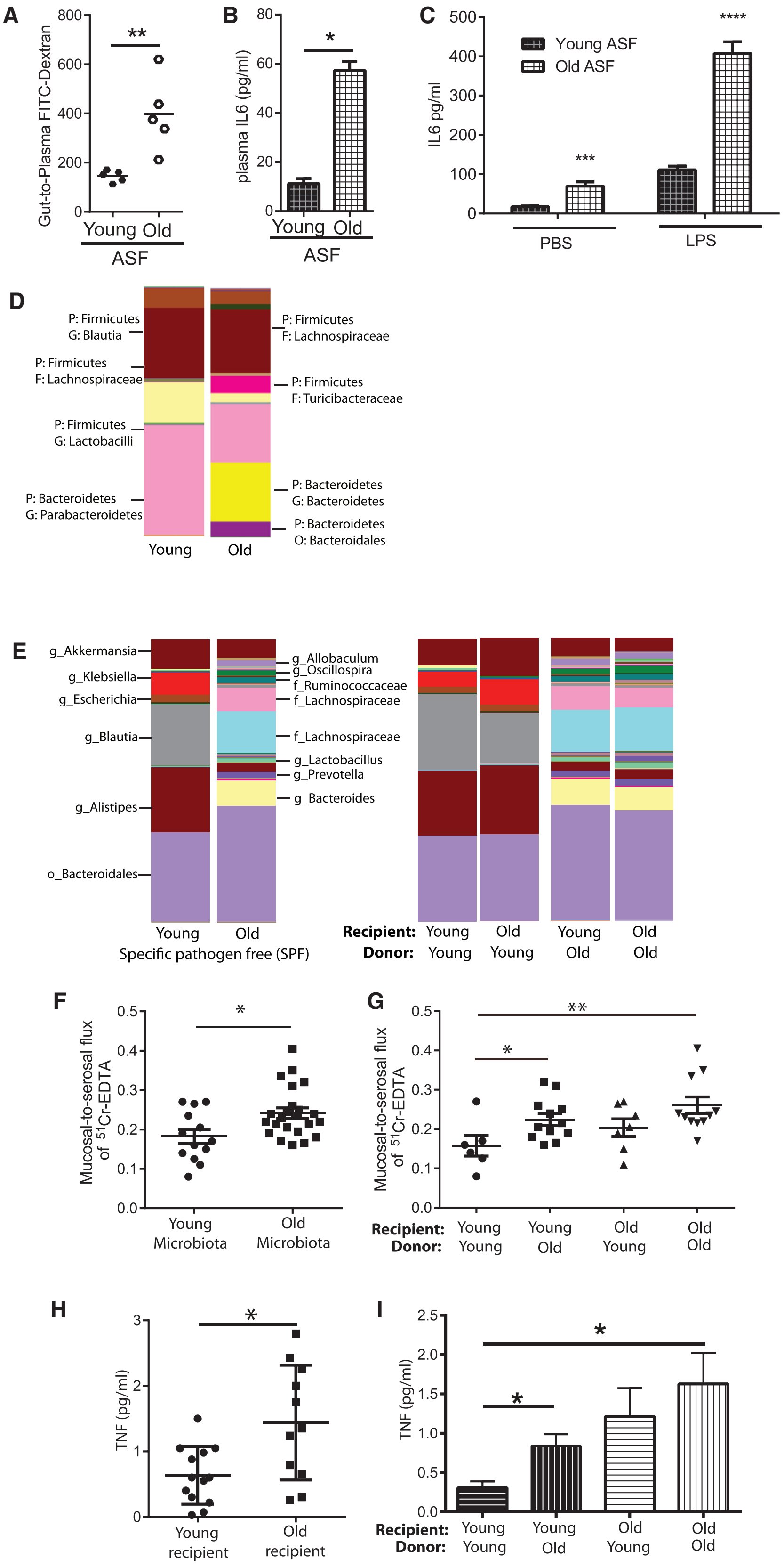

Figure 4. Age-Associated Inflammation Is Dependent on the Composition of the Intestinal Microbial Community

(A and B) Mice with minimal ASF-derived microbiota were aged, and their intestinal permeability was measured via FITC-dextran oral gavage assay (A; n = 5). Old (18 months) ASF mice had higher intestinal permeability than young (10–14 weeks) ASF mice in addition to higher levels of plasma IL6 (B).

(C) IL6 production after LPS stimulation in whole blood was higher in old ASF mice (n = 3).

(D) The taxa summary of microbiota of young and old ASF mice indicates that age-related microbial dysbiosis occurs.

(E) Taxa summaries illustrate that the composition of the young and old microbiota is retained upon transfer to young or old GF mice (n = 5–16 mice/group over four independent colonization experiments).

(F) Paracellular permeability was measured in GF mice colonized with young and old microbiota (n = 6–8 mice per group). There was a statistically significant increase in paracellular permeability in mice colonized with old microbiota (n = 23 total, n = 11 young mice and n = 12 old mice) compared to young microbiota (n = 13, n = 6 young mice and n = 7 old mice). This demonstrates that the age of the microbiota alters barrier function.

(G) Colonization of young GF mice with old microbiota increased paracellular permeability compared to those colonized with young microbiota; however, old mice colonized with either young or old microbiota demonstrated increased permeability, indicating that age-related changes in the host increased susceptibility to the microbiota.

(H) Circulating TNF was measured from all the young GF mice (n = 13 total, n = 5 colonized with young microbiota and n = 8 colonized with old microbiota) and old GF mice (n = 11 total, n = 5 colonized with the young microbiota and n = 6 colonized with the old microbiota). Old recipient mice had higher levels of circulating TNF than young recipient mice.

(I) The microbiota contributed to the increased TNF in the circulation of young mice, since young GF mice colonized with old microbiota had higher circulating levels of TNF than those colonized with young microbiota. In contrast, colonization with either the young or old microbiota increased circulating TNF in old GF mice, indicating that the age of the host interacts with the age of the microbiota to induce systemic inflammation. Bars represent the mean ± SEM. Statistical significance was determined using the Mann-Whitney test or two-way ANOVA with Fisher's post-test or unpaired t test where appropriate (*$p < 0.05$, **$p < 0.005$, and ***$p < 0.0005$).

Table 1. OTUs that Were Significantly Changed in Old SPF and TNF KO Mice

Family	Genus	Number of OTUs (WT)[a]	Old > Young (WT)	Old > Young (TNF KO)	Number of OTUs (TNF KO)
Increased					
Ruminococcaceae	*Ruminococcus*	6	****	NS	7
Lachnospiraceae	*Clostridium*	34	****	**[b]	34
Ruminococcaceae	*Clostridium*	14	****	NS	16
Prevotellaceae	*Prevotella*	39	**	**	44
Erysipelotrichaceae	*Allobaculum*	54	****	*	60
Lachnospiraceae	many identified	354	*	*[b]	343
Bifidobacteriaceae	*Bifidobacterium*	17	***	**	17
Ruminococcaceae	*Oscillospira*	54	****	NS	56
Lactobacillaceae	*Lactobacillus*	53	***	****[b]	57
Bacteroidaceae	*Bacteroides*	52	**	***	71
Coriobacteriaceae	*Adlercreutzia*	22	***	NS	23
Peptococcaceae	not identified	14	*	NS	15
Catabacteriaceae	not identified	51	****	**[b]	65
Coriobacteriaceae	not identified	14	***	NS	14
Decreased					
Family	Genus	Number of OTUs (WT)	Old < Young (WT)	Old < Young (TNF KO)	Number of OTUs (TNF KO)
Rikenellaceae	*Alistipes*	48	**	NS	52
Verrucomicrobiaceae	*Akkermansia*	41	****	*[c]	49
Lachnospiraceae	*Blautia*	11	****	*[c]	10

*p < 0.05, **p < 0.005, ***p < 0.0005, and ****p < 0.0001; NS, not significant.
[a]Number of OTUs is the number of OTUs found in the samples that are attributed to the family or genus.
[b]OTUs that were significantly decreased in old TNF KO mice.
[c]OTUs that were significantly increased in old TNF KO mice.

whether the fecal microbiota of TNF KO mice were less divergent with age than the WT microbiota, the distances between young-old pairs were calculated using the Bray-Curtis distance matrix. Average distances between the young and old WT mice were greater than the young and old TNF KO mice, indicating that the beta diversity was greater between young and old WT mice ($p = 0.04$, Mann-Whitney test). The divergence in the microbiota that occurs in aging WT mice and the decreased divergence that occurs in aging TNF KO mice is visualized in Figure 5C and listed in Table 1.

To further evaluate the potential of TNF to induce changes to the intestinal microbiota, young and old WT mice were treated with the anti-TNF drug Humira for 2 weeks, which reduced TNF levels in old mice to below the limit of detection. Anti-TNF, but not an IgG control, altered the composition of the intestinal microbiota of old mice (Figure 5D), as there was a significant ($p = 0.045$) interaction on the Bray-Curtis distances of the old Humira and IgG microbiota, but not the young Humira- and IgG-treated microbiota. This demonstrates that the microbiota can be manipulated by altering the inflammatory status of the host. Changes in specific OTUs during anti-TNF treatment are described in Table 2. Despite altering the microbiota, anti-TNF treatment had no measurable effect on intestinal permeability, as measured by translocation of FITC-dextran (data not shown), indicating that these changes to the composition of the intestinal microbiota were not a consequence of altered TNF levels having any direct effect on intestinal permeability. These experiments do not rule out a role for age-associated increases in TNF driving intestinal permeability by directly altering intestinal barrier function, but they do indicate that elevated levels of TNF contribute to age-associated microbial dysbiosis. Collectively these data are most consistent with dysbiosis causing increased intestinal permeability and translocation of bacterial products, which increases systemic inflammation that ultimately impairs macrophage function, a model for which is presented in Figure 5E.

DISCUSSION

Age-associated inflammation is a strong risk factor for overall mortality in older adults. In fact, individuals having higher than age-average levels of inflammatory markers are more likely to be hospitalized (de Gonzalo-Calvo et al., 2012a), have higher all-cause mortality rates (Giovannini et al., 2011), be frail (Leng et al., 2007), be less independent (de Gonzalo-Calvo et al., 2012b), and are more likely to have a variety of late-life diseases (Bruunsgaard, 2006; Bruunsgaard et al., 1999) Age-associated inflammation has also been shown to increase susceptibility to pneumococcal infection (Yende et al., 2005, 2013), and it is associated with increased disease severity and decreased survival from pneumococcal infection in older adults (Antunes et al., 2002; Reade et al., 2009). Despite the clinical importance of age-associated inflammation, the etiological factors that lead to its development have not been identified. This study demonstrates that age-associated inflammation and microbial dysbiosis drive intestinal permeability and translocation of bacterial

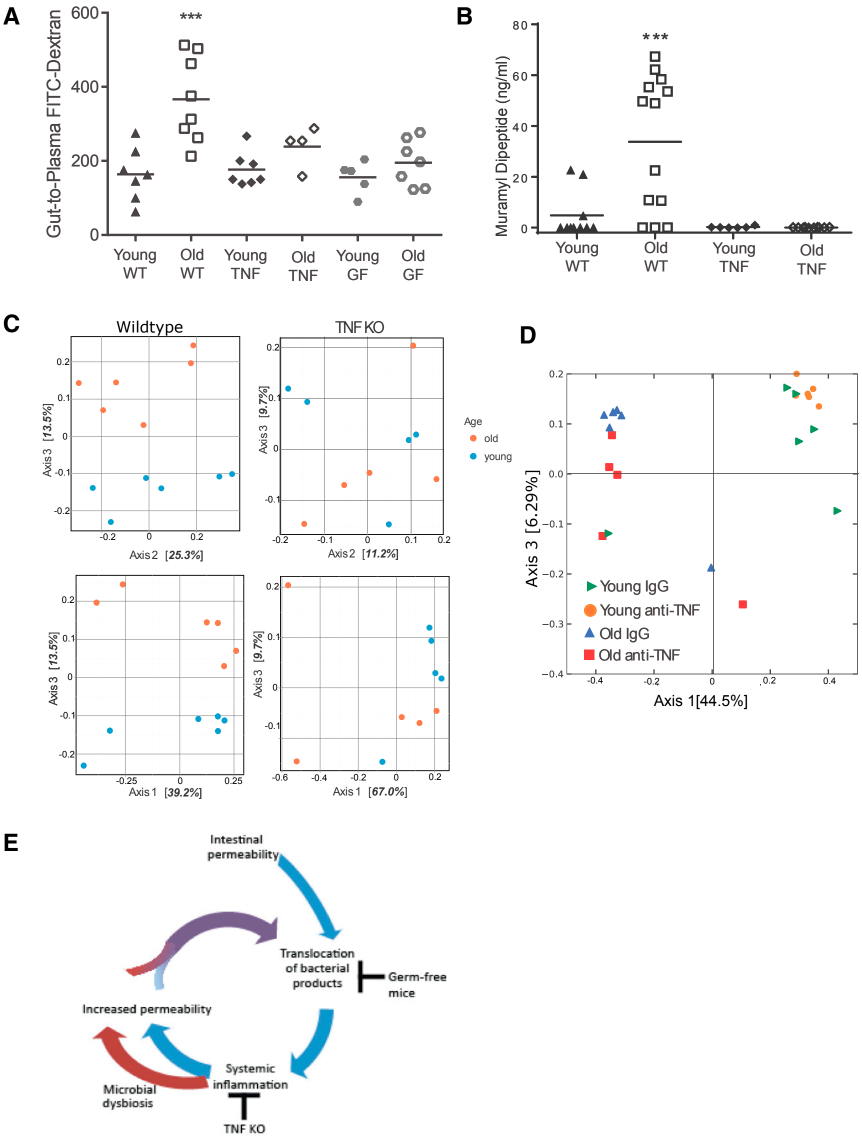

Figure 5. Microbial Dysbiosis Occurs with Age and Inflammation

(A) Intestinal permeability, as measured by plasma FITC-dextran following oral gavage, was increased in old WT/SPF mice, but not old TNF KO or old GF mice.

(B) Circulating MDP is increased in old SPF/WT mice. Old TNF KO and GF mice are not significantly different from young WT/SPF mice (n = 5–10). GF mice do not have any detectable MDP in the circulation.

(C) Principal coordinate analysis based on Bray-Curtis demonstrates that the microbial communities of old WT mice diverge from young mice, but this is not the case in old TNF KO mice. Mice were sampled from multiple cages. Chi-square of the likelihood ratio test in DESeq2 shows old and young microbiota are significantly different ($p < 0.001$).

(D) Anti-TNF (Adalimumab) or a human IgG isotype control was administered at a dose of 50 ng/g of body weight every other day for 2 weeks. Principal co-ordinate analysis was used to visualize differences in the microbial communities after 2 weeks of anti-TNF treatment. Anti-TNF treatment altered the composition of the fecal microbiota of old, but not young, mice.

(E) Basal translocation of microbial products occurs throughout life; however, with age, these induce an inflammatory response, which contributes to microbial dysbiosis. Microbial dysbiosis increases intestinal permeability, which increases bacterial translocation. This feed-forward process increases with age.

components, further fueling inflammation and impairing cellular antibacterial function.

In humans, transient endotoxemia occurs naturally after ingestion of high-fat meals, vigorous exercise, and in many diseases (Kelly et al., 2012). Microbial translocation has been shown to occur in HIV patients due to a loss of immune control at the gut mucosa, and this translocation leads to a state of immune activation and systemic inflammation that is reminiscent of what is observed in normal aging (Brenchley et al., 2006). This increase in chronic inflammation correlates with early mortality, which is often due to premature development of diseases of age such as cardiovascular disease (Sandler et al., 2011). In simian models of HIV, translocation of bacterial products is a precursor of immune activation and macrophage dysfunction (Estes et al., 2010). In these models, reducing levels of circulating LPS by chelation with the drug sevelamer prevents immune dysfunction, systemic inflammation, and, most relevant to our study, reduces intestinal permeability, implying that bacterial translocation and subsequent inflammation are a driver of intestinal permeability, rather than a readout of intestinal damage (Kristoff et al., 2014). These data are consistent with our model in which both GF and TNF KO mice (which do not have increased levels of circulating bacterial products) are protected from age-associated inflammation. Unlike the GF mice, which are protected by virtue of not being exposed to bacteria, the TNF mice may be protected because they do not undergo microbial dysbiosis with age, which we demonstrate confers intestinal permeability and systemic inflammation in the context of the aged host. This may be an evolutionarily conserved component of the aging process, since intestinal permeability has been demonstrated to precede systemic inflammation and to be a marker of premature death in *Drosophila* (Rera et al., 2012).

Metchnikoff made careful observations that, in acute inflammation, macrophage-mediated phagocytosis seemed to be impaired. In examining autopsy samples of the elderly, he noticed that brain tissue macrophages seemed to be associated with areas of damage, and he hypothesized that their presence might do more harm than good. He also observed that the integrity of the gut changed with age and concluded that, "it is indubitable, therefore, that the intestinal microbes or their poisons may reach the system generally and bring harm to it" (Metchnikoff, 1907). He believed that this macrophage "intoxification" had

Table 2. OTUs Altered by Anti-TNF Treatment in Old Mice

Increased			
Family	Genus	Old SPF > Young SPF	Decreased by Anti-TNF Treatment
Erysipelotrichaceae	not identified	****	NS
Ruminococcaceae	*Ruminococcus*	****	NS
Lachnospiraceae	*Clostridium*	****	NS
Ruminococcaceae	*Clostridium*	****	NS
Prevotellaceae	*Prevotella*	**	NS
Erysipelotrichaceae	*Allobaculum*	****	NS
Lachnospiraceae	many identified	*	NS
Bifidobacteriaceae	*Bifidobacterium*	***	NS
Ruminococcaceae	*Oscillospira*	****	NS
Lactobacillaceae	*Lactobacillus*	***	NS
Bacteroidaceae	*Bacteroides*	**	*
Coriobacteriaceae	*Adlercreutzia*	***	*
Peptococcaceae	not identified	*	**
Catabacteriaceae	not identified	****	* (increased)
Coriobacteriaceae	not identified	***	*
Ruminococcaceae	*Subdoligranulum*	NS	*
Decreased			
Family	Genus	Old < Young	Increased by Anti-TNF Treatment
Rikenellaceae	*Alistipes*	**	NS
Verrucomicrobiaceae	*Akkermansia*	****	NS
Lachnospiraceae	*Blautia*	****	NS
Lachnospiraceae	*Roseburia*	NS	*
Eubacteriaceae	*Anaerofustis*	NS	*

Families that are higher in old SPF mice are listed. Those that are decreased by anti-TNF treatment in old mice are labeled with an asterisk. *$p < 0.05$, **$p < 0.005$, ***$p < 0.0005$, and ****$p < 0.0001$; NS, not significant.

systemic effects and led to deterioration of even distal tissues. Our observations are consistent with his, as we observed an increase in circulating bacterial products as our WT mice aged and evidence of systemic and distal inflammation. Although we only measured the presence of bacterial products in the serum, it is entirely possible that they also enter the lymphatics. It has recently been demonstrated that acute infections can permanently remodel the lymphatics, causing them to become more permeable (Fonseca et al., 2015). Whether age-related microbial dysbiosis increases lymphatic permeability is unknown. Regardless of how bacterial products enter the periphery, the systemic inflammation they cause has profound effects on myelopoiesis, since macrophages derived from bone marrow precursors in the absence of the aging microenvironment become hyper-inflammatory and have poor killing capacity. Although Metchnikoff imagined that loss of macrophage function was a result of age-associated inflammation, he did not predict that they may also contribute to the global inflammatory state. In fact, it appears as though both aged monocytes (Puchta et al., 2016) and macrophages (Mirsoian et al., 2014) contribute to chronic inflammation, as their depletion reduces levels of inflammatory cytokines.

Consistent with our findings that the gut microbiota can also influence systemic (i.e., lung) inflammation and tissue damage, it has been shown that increased circulating bacterial toxins result in reduced tight junction gene expression and lethal pulmonary damage following fecal transplantation (Ji et al., 2014). The authors suggest that these changes may occur following overgrowth of gut microbes and/or threshold production of bacterial products, resulting in their systemic translocation, increased inflammation, and ensuing pulmonary endothelial damage. The bacterial taxa that were mainly implicated in this pathogenicity were members of Clostridia, which others have also demonstrated have distinct abundance patterns in the aging gut microbial community (Claesson et al., 2011, 2012).

Although it has been suggested that changes in the microbiota might drive the ills of aging, determining cause and effect has been challenging. Numerous studies have demonstrated that there are characteristic changes in gut microbial communities in elderly humans (Claesson et al., 2011; Mäkivuokko et al., 2010; Mariat et al., 2009; Zwielehner et al., 2009) and that these changes correlate with health status in the elderly population (Bartosch et al., 2004; Claesson et al., 2012). Furthermore, therapeutic manipulation of the gut microbiota appears to improve immune function in the elderly. For example, oral supplementation with *Bifidobacterium* increased lymphocyte proportions in the circulation, improved the anti-tumoricidal activity of natural killer cells, and restored phagocytosis in peripheral blood mononuclear cells and neutrophils (Gill et al., 2001a, 2001b). Interestingly, these benefits were most strongly evident in individuals 70 years of age and older, as well as those individuals who demonstrated the greatest degree of cellular immunosenescence. Furthermore, dysbiosis in HIV patients, which shows many

parallels to that which occurs in the elderly (including decreased *Bifidobacteria* frequency and increased clusters of *Clostridium*), decreases following prebiotic administration. This led to a decrease in the overall degree of microbial translocation and ultimately improved immune cell function (Gori et al., 2011). The microbial communities of the elderly gut appear to be strongly influenced by diet (Claesson et al., 2012), and dietary interventions designed to restore a robust microbiota may improve anti-bacterial immunity by reducing age-associated inflammation and macrophage immunosenescence (Clements and Carding, 2016; Vaiserman et al., 2017).

Although manipulation of the microbiota may improve health in the elderly, until now it has not been clear whether microbial dysbiosis is a driver of immune dysfunction. For example, it has been demonstrated that gut microbial composition correlates with levels of circulating cytokines and markers of health in the elderly (Claesson et al., 2012) and that intestinal permeability and systemic inflammation increase in old mice (Scott et al., 2017), but not whether the microbiota drive these changes. Our data demonstrate that microbial dysbiosis occurs with age, even in minimal microbiota, and these changes are sufficient to promote age-associated inflammation, although we have not determined whether this is due to enrichment of specific species, changes in microbe-microbe interactions, alterations in the functional capacity of the aging microbiota (e.g., changes in short-chain fatty acid production), or loss of compartmentalization of the microbiota as is found in *Drosophila* (Li et al., 2016). Interestingly there may be a causal relationship between age-associated inflammation and microbial dysbiosis, since we found that TNF KO mice had a less divergent microbiota with age and treatment with anti-TNF altered the microbial communities of aged mice. Although there were significant changes in the composition of the microbiota with anti-TNF treatment, we have not yet identified which members of the microbial community alter barrier function with age. Further experiments will need to be performed to determine if it is the loss of beneficial members of the microbial community, overgrowth of harmful members, or a shift in metabolism that contributes to this phenomenon.

Metchnikoff had great faith that the appropriate experiments could be performed to demonstrate that manipulation of the intestinal microbiota would extend life. Until that time he suggested, "… those who wish to preserve their intelligence as long as possible and to make their cycle of life as complete and as normal as possible under present conditions, must depend on general sobriety and on habits conforming to the rules of rational hygiene." The experiments he envisioned remain to be performed, and, until they are, the only reliable ways to reduce age-associated inflammation, delay the onset of inflammatory diseases, and prolong life are a sensible diet (Fontana and Hu, 2014; Fontana and Klein, 2007) and exercise (Lee et al., 1995). For those of us less inclined to live a lifestyle of general sobriety, targeting age-associated inflammation may provide an attractive alternative.

STAR★METHODS

Detailed methods are provided in the online version of this paper and include the following:

- KEY RESOURCES TABLE
- CONTACT FOR REAGENT AND RESOURCE SHARING
- EXPERIMENTAL MODEL AND SUBJECT DETAILS
  - Ethics statement
  - Mouse Experiments
- METHOD DETAILS
  - TNF ablation
  - Histological analysis
  - Measurement of cytokine production
  - Macrophage culture
  - Bacterial killing assays
  - In vitro and in vivo permeability
  - MDP Detection Bioassay
  - Germ-free Mouse Recolonization
  - Bacterial profiling by deep sequencing analysis of 16S rRNA with Illumina
- QUANTIFICATION AND STATISTICAL ANALYSIS
- DATA AND SOFTWARE AVAILABILITY

SUPPLEMENTAL INFORMATION

Supplemental Information includes one figure and can be found with this article online at http://dx.doi.org/10.1016/j.chom.2017.03.002.

AUTHOR CONTRIBUTIONS

Conceptualization, D.M.E.B., M.G.S., E.F.V., and D.J.D.; Methodology, J.D.S., E.F.V., and M.G.S.; Investigation, N.T., A.P., A.N., C.S., J.C.S., C.P.V., D.L., L.P.S., J.J., and K.P.F.; Resources, M.J.L.; Review & Editing, D.M.E.B., M.G.S., and D.J.D.; Supervision, D.M.E.B., M.G.S., J.D.S., and E.F.V.

ACKNOWLEDGMENTS

A.P. was supported by an Ontario Graduate Scholarship. C.P.V. was supported by a fellowship from the Canadian Thoracic Society. A.N. was supported by a scholarship from the Canadian Institutes of Health Research (CIHR). N.T. was supported by an Early Researcher Award from the Ontario Ministry of Research and Innovation. This work was funded by grants from the CIHR to D.M.E.B. (FRN 123404 and 224026). E.F.V. is supported by the CIHR (MOP 142773). E.F.V., M.G.S., and D.M.E.B. are supported by the CIHR and hold Canada Research Chairs. Work in the Bowdish laboratory is supported by the McMaster Immunology Research Centre (MIRC) and the M.G. DeGroote Institute for Infectious Disease Research (IIDR). D.J.D. is an MRC Senior Research Fellow (G1002046). The authors would like to thank Kate Manners and Laura Rossi for isolation and preparation of DNA for microbiome analysis and Fiona Whelan for advice on microbiome analysis.

Received: October 4, 2016
Revised: February 1, 2017
Accepted: March 2, 2017
Published: April 12, 2017

REFERENCES

Antunes, G., Evans, S.A., Lordan, J.L., and Frew, A.J. (2002). Systemic cytokine levels in community-acquired pneumonia and their association with disease severity. Eur. Respir. J. *20*, 990–995.

Bartosch, S., Fite, A., Macfarlane, G.T., and McMurdo, M.E. (2004). Characterization of bacterial communities in feces from healthy elderly volunteers and hospitalized elderly patients by using real-time PCR and effects of antibiotic treatment on the fecal microbiota. Appl. Environ. Microbiol. *70*, 3575–3581.

Bartram, A.K., Lynch, M.D., Stearns, J.C., Moreno-Hagelsieb, G., and Neufeld, J.D. (2011). Generation of multimillion-sequence 16S rRNA gene libraries from

complex microbial communities by assembling paired-end illumina reads. Appl. Environ. Microbiol. *77*, 3846–3852.

Bouchlaka, M.N., Sckisel, G.D., Chen, M., Mirsoian, A., Zamora, A.E., Maverakis, E., Wilkins, D.E., Alderson, K.L., Hsiao, H.H., Weiss, J.M., et al. (2013). Aging predisposes to acute inflammatory induced pathology after tumor immunotherapy. J. Exp. Med. *210*, 2223–2237.

Brenchley, J.M., Price, D.A., Schacker, T.W., Asher, T.E., Silvestri, G., Rao, S., Kazzaz, Z., Bornstein, E., Lambotte, O., Altmann, D., et al. (2006). Microbial translocation is a cause of systemic immune activation in chronic HIV infection. Nat. Med. *12*, 1365–1371.

Bruunsgaard, H. (2006). The clinical impact of systemic low-level inflammation in elderly populations. With special reference to cardiovascular disease, dementia and mortality. Dan. Med. Bull. *53*, 285–309.

Bruunsgaard, H., Andersen-Ranberg, K., Jeune, B., Pedersen, A.N., Skinhøj, P., and Pedersen, B.K. (1999). A high plasma concentration of TNF-alpha is associated with dementia in centenarians. J. Gerontol. A Biol. Sci. Med. Sci. *54*, M357–M364.

Caporaso, J.G., Kuczynski, J., Stombaugh, J., Bittinger, K., Bushman, F.D., Costello, E.K., Fierer, N., Peña, A.G., Goodrich, J.K., Gordon, J.I., et al. (2010). QIIME allows analysis of high-throughput community sequencing data. Nat. Methods *7*, 335–336.

Claesson, M.J., Cusack, S., O'Sullivan, O., Greene-Diniz, R., de Weerd, H., Flannery, E., Marchesi, J.R., Falush, D., Dinan, T., Fitzgerald, G., et al. (2011). Composition, variability, and temporal stability of the intestinal microbiota of the elderly. Proc. Natl. Acad. Sci. USA *108* (*Suppl 1*), 4586–4591.

Claesson, M.J., Jeffery, I.B., Conde, S., Power, S.E., O'Connor, E.M., Cusack, S., Harris, H.M., Coakley, M., Lakshminarayanan, B., O'Sullivan, O., et al. (2012). Gut microbiota composition correlates with diet and health in the elderly. Nature *488*, 178–184.

Clark, R.I., Salazar, A., Yamada, R., Fitz-Gibbon, S., Morselli, M., Alcaraz, J., Rana, A., Rera, M., Pellegrini, M., Ja, W.W., and Walker, D.W. (2015). Distinct Shifts in Microbiota Composition during Drosophila Aging Impair Intestinal Function and Drive Mortality. Cell Rep. *12*, 1656–1667.

Clements, S.J., and Carding, S.R. (2016). Diet, the intestinal microbiota and immune health in ageing. Crit. Rev. Food Sci. Nutr.

Conley, M.N., Wong, C.P., Duyck, K.M., Hord, N., Ho, E., and Sharpton, T.J. (2016). Aging and serum MCP-1 are associated with gut microbiome composition in a murine model. PeerJ *4*, e1854.

de Gonzalo-Calvo, D., de Luxán-Delgado, B., Martínez-Camblor, P., Rodríguez-González, S., García-Macia, M., Suárez, F.M., Solano, J.J., Rodríguez-Colunga, M.J., and Coto-Montes, A. (2012a). Chronic inflammation as predictor of 1-year hospitalization and mortality in elderly population. Eur. J. Clin. Invest. *42*, 1037–1046.

de Gonzalo-Calvo, D., de Luxán-Delgado, B., Rodríguez-González, S., García-Macia, M., Suárez, F.M., Solano, J.J., Rodríguez-Colunga, M.J., and Coto-Montes, A. (2012b). Interleukin 6, soluble tumor necrosis factor receptor I and red blood cell distribution width as biological markers of functional dependence in an elderly population: a translational approach. Cytokine *58*, 193–198.

Dewhirst, F.E., Chien, C.C., Paster, B.J., Ericson, R.L., Orcutt, R.P., Schauer, D.B., and Fox, J.G. (1999). Phylogeny of the defined murine microbiota: altered Schaedler flora. Appl. Environ. Microbiol. *65*, 3287–3292.

Estes, J.D., Harris, L.D., Klatt, N.R., Tabb, B., Pittaluga, S., Paiardini, M., Barclay, G.R., Smedley, J., Pung, R., Oliveira, K.M., et al. (2010). Damaged intestinal epithelial integrity linked to microbial translocation in pathogenic simian immunodeficiency virus infections. PLoS Pathog. *6*, e1001052.

Fonseca, D.M., Hand, T.W., Han, S.J., Gerner, M.Y., Glatman Zaretsky, A., Byrd, A.L., Harrison, O.J., Ortiz, A.M., Quinones, M., Trinchieri, G., et al. (2015). Microbiota-Dependent Sequelae of Acute Infection Compromise Tissue-Specific Immunity. Cell *163*, 354–366.

Fontana, L., and Hu, F.B. (2014). Optimal body weight for health and longevity: bridging basic, clinical, and population research. Aging Cell *13*, 391–400.

Fontana, L., and Klein, S. (2007). Aging, adiposity, and calorie restriction. JAMA *297*, 986–994.

Franceschi, C., Bonafè, M., Valensin, S., Olivieri, F., De Luca, M., Ottaviani, E., and De Benedictis, G. (2000). Inflamm-aging. An evolutionary perspective on immunosenescence. Ann. N Y Acad. Sci. *908*, 244–254.

Franceschi, C., Capri, M., Monti, D., Giunta, S., Olivieri, F., Sevini, F., Panourgia, M.P., Invidia, L., Celani, L., Scurti, M., et al. (2007). Inflammaging and anti-inflammaging: a systemic perspective on aging and longevity emerged from studies in humans. Mech. Ageing Dev. *128*, 92–105.

Gill, H.S., Rutherfurd, K.J., and Cross, M.L. (2001a). Dietary probiotic supplementation enhances natural killer cell activity in the elderly: an investigation of age-related immunological changes. J. Clin. Immunol. *21*, 264–271.

Gill, H.S., Rutherfurd, K.J., Cross, M.L., and Gopal, P.K. (2001b). Enhancement of immunity in the elderly by dietary supplementation with the probiotic Bifidobacterium lactis HN019. Am. J. Clin. Nutr. *74*, 833–839.

Giovannini, S., Onder, G., Liperoti, R., Russo, A., Carter, C., Capoluongo, E., Pahor, M., Bernabei, R., and Landi, F. (2011). Interleukin-6, C-reactive protein, and tumor necrosis factor-alpha as predictors of mortality in frail, community-living elderly individuals. J. Am. Geriatr. Soc. *59*, 1679–1685.

Gori, A., Rizzardini, G., Van't Land, B., Amor, K.B., van Schaik, J., Torti, C., Quirino, T., Tincati, C., Bandera, A., Knol, J., et al. (2011). Specific prebiotics modulate gut microbiota and immune activation in HAART-naive HIV-infected adults: results of the "COPA" pilot randomized trial. Mucosal Immunol. *4*, 554–563.

Hinojosa, E., Boyd, A.R., and Orihuela, C.J. (2009). Age-associated inflammation and toll-like receptor dysfunction prime the lungs for pneumococcal pneumonia. J. Infect. Dis. *200*, 546–554.

Ji, Y., Sun, S., Goodrich, J.K., Kim, H., Poole, A.C., Duhamel, G.E., Ley, R.E., and Qi, L. (2014). Diet-induced alterations in gut microflora contribute to lethal pulmonary damage in TLR2/TLR4-deficient mice. Cell Rep. *8*, 137–149.

Kelly, C.J., Colgan, S.P., and Frank, D.N. (2012). Of microbes and meals: the health consequences of dietary endotoxemia. Nutr. Clin. Pract. *27*, 215–225.

Kristoff, J., Haret-Richter, G., Ma, D., Ribeiro, R.M., Xu, C., Cornell, E., Stock, J.L., He, T., Mobley, A.D., Ross, S., et al. (2014). Early microbial translocation blockade reduces SIV-mediated inflammation and viral replication. J. Clin. Invest. *124*, 2802–2806.

Lee, I.M., Hsieh, C.C., and Paffenbarger, R.S., Jr. (1995). Exercise intensity and longevity in men. The Harvard Alumni Health Study. JAMA *273*, 1179–1184.

Leng, S.X., Xue, Q.L., Tian, J., Walston, J.D., and Fried, L.P. (2007). Inflammation and frailty in older women. J. Am. Geriatr. Soc. *55*, 864–871.

Li, H., Qi, Y., and Jasper, H. (2016). Preventing Age-Related Decline of Gut Compartmentalization Limits Microbiota Dysbiosis and Extends Lifespan. Cell Host Microbe *19*, 240–253.

Mäkivuokko, H., Tiihonen, K., Tynkkynen, S., Paulin, L., and Rautonen, N. (2010). The effect of age and non-steroidal anti-inflammatory drugs on human intestinal microbiota composition. Br. J. Nutr. *103*, 227–234.

Mariat, D., Firmesse, O., Levenez, F., Guimarães, V., Sokol, H., Doré, J., Corthier, G., and Furet, J.P. (2009). The Firmicutes/Bacteroidetes ratio of the human microbiota changes with age. BMC Microbiol. *9*, 123.

Martin, M. (2011). Cutadapt removes adapter sequences from high-throughput sequencing reads. EMBnet.journal *17*, 10–12.

Masella, A.P., Bartram, A.K., Truszkowski, J.M., Brown, D.G., and Neufeld, J.D. (2012). PANDAseq: paired-end assembler for illumina sequences. BMC Bioinformatics *13*, 31.

McMurdie, P.J., and Holmes, S. (2013). phyloseq: an R package for reproducible interactive analysis and graphics of microbiome census data. PLoS ONE *8*, e61217.

Metchnikoff, E. (1907). The Prolongation of Life: Optimistic Studies (G. P. Putnam's Sons).

Mirsoian, A., Bouchlaka, M.N., Sckisel, G.D., Chen, M., Pai, C.C., Maverakis, E., Spencer, R.G., Fishbein, K.W., Siddiqui, S., Monjazeb, A.M., et al. (2014). Adiposity induces lethal cytokine storm after systemic administration of stimulatory immunotherapy regimens in aged mice. J. Exp. Med. *211*, 2373–2383.

Noth, R., Stüber, E., Häsler, R., Nikolaus, S., Kühbacher, T., Hampe, J., Bewig, B., Schreiber, S., and Arlt, A. (2012). Anti-TNF-α antibodies improve intestinal barrier function in Crohn's disease. J. Crohn's Colitis *6*, 464–469.

Novakowski, K.E., Loukov, D., Chawla, V., and Bowdish, D.M. (2017). Bacterial Binding, Phagocytosis, and Killing: Measurements Using Colony Forming Units. Methods Mol. Biol. *1519*, 297–309.

Puchta, A., Naidoo, A., Verschoor, C.P., Loukov, D., Thevaranjan, N., Mandur, T.S., Nguyen, P.S., Jordana, M., Loeb, M., Xing, Z., et al. (2016). TNF Drives Monocyte Dysfunction with Age and Results in Impaired Anti-pneumococcal Immunity. PLoS Pathog. *12*, e1005368.

Reade, M.C., Yende, S., D'Angelo, G., Kong, L., Kellum, J.A., Barnato, A.E., Milbrandt, E.B., Dooley, C., Mayr, F.B., Weissfeld, L., and Angus, D.C.; Genetic and Inflammatory Markers of Sepsis Investigators (2009). Differences in immune response may explain lower survival among older men with pneumonia. Crit. Care Med. *37*, 1655–1662.

Rera, M., Clark, R.I., and Walker, D.W. (2012). Intestinal barrier dysfunction links metabolic and inflammatory markers of aging to death in Drosophila. Proc. Natl. Acad. Sci. USA *109*, 21528–21533.

Sandler, N.G., Wand, H., Roque, A., Law, M., Nason, M.C., Nixon, D.E., Pedersen, C., Ruxrungtham, K., Lewin, S.R., Emery, S., et al.; INSIGHT SMART Study Group (2011). Plasma levels of soluble CD14 independently predict mortality in HIV infection. J. Infect. Dis. *203*, 780–790.

Scott, K.A., Ida, M., Peterson, V.L., Prenderville, J.A., Moloney, G.M., Izumo, T., Murphy, K., Murphy, A., Ross, R.P., Stanton, C., et al. (2017). Revisiting Metchnikoff: Age-related alterations in microbiota-gut-brain axis in the mouse. Brain Behav. Immun., S0889-1591(17)30034-X.

Slack, E., Hapfelmeier, S., Stecher, B., Velykoredko, Y., Stoel, M., Lawson, M.A., Geuking, M.B., Beutler, B., Tedder, T.F., Hardt, W.D., et al. (2009). Innate and adaptive immunity cooperate flexibly to maintain host-microbiota mutualism. Science *325*, 617–620.

Söderholm, J.D., Streutker, C., Yang, P.C., Paterson, C., Singh, P.K., McKay, D.M., Sherman, P.M., Croitoru, K., and Perdue, M.H. (2004). Increased epithelial uptake of protein antigens in the ileum of Crohn's disease mediated by tumour necrosis factor alpha. Gut *53*, 1817–1824.

Stearns, J.C., Davidson, C.J., McKeon, S., Whelan, F.J., Fontes, M.E., Schryvers, A.B., Bowdish, D.M., Kellner, J.D., and Surette, M.G. (2015). Culture and molecular-based profiles show shifts in bacterial communities of the upper respiratory tract that occur with age. ISME J. *9*, 1246–1259.

Ubeda, C., Lipuma, L., Gobourne, A., Viale, A., Leiner, I., Equinda, M., Khanin, R., and Pamer, E.G. (2012). Familial transmission rather than defective innate immunity shapes the distinct intestinal microbiota of TLR-deficient mice. J. Exp. Med. *209*, 1445–1456.

Vaiserman, A.M., Koliada, A.K., and Marotta, F. (2017). Gut microbiota: A player in aging and a target for anti-aging intervention. Ageing Res. Rev. *35*, 36–45.

Verdu, E.F., Huang, X., Natividad, J., Lu, J., Blennerhassett, P.A., David, C.S., McKay, D.M., and Murray, J.A. (2008). Gliadin-dependent neuromuscular and epithelial secretory responses in gluten-sensitive HLA-DQ8 transgenic mice. Am. J. Physiol. Gastrointest. Liver Physiol. *294*, G217–G225.

Verschoor, C.P., Naidoo, A., Wallace, J.G., Johnstone, J., Loeb, M., Bramson, J.L., and Bowdish, D.M. (2015). Circulating muramyl dipeptide is negatively associated with interleukin-10 in the frail elderly. Inflammation *38*, 272–277.

Whelan, F.J., Verschoor, C.P., Stearns, J.C., Rossi, L., Luinstra, K., Loeb, M., Smieja, M., Johnstone, J., Surette, M.G., and Bowdish, D.M. (2014). The loss of topography in the microbial communities of the upper respiratory tract in the elderly. Ann. Am. Thorac. Soc. *11*, 513–521.

Ye, Y. (2011). Identification and quantification of abundant species from pyrosequences of 16S rRNA by consensus alignment. Proceedings (IEEE Int. Conf. Bioinformatics Biomed.) *2010*, 153–157.

Yende, S., Tuomanen, E.I., Wunderink, R., Kanaya, A., Newman, A.B., Harris, T., de Rekeneire, N., and Kritchevsky, S.B. (2005). Preinfection systemic inflammatory markers and risk of hospitalization due to pneumonia. Am. J. Respir. Crit. Care Med. *172*, 1440–1446.

Yende, S., Alvarez, K., Loehr, L., Folsom, A.R., Newman, A.B., Weissfeld, L.A., Wunderink, R.G., Kritchevsky, S.B., Mukamal, K.J., London, S.J., et al.; Atherosclerosis Risk in Communities Study; Cardiovascular Health Study; Health, Aging, and Body Composition Study (2013). Epidemiology and long-term clinical and biologic risk factors for pneumonia in community-dwelling older Americans: analysis of three cohorts. Chest *144*, 1008–1017.

Zwielehner, J., Liszt, K., Handschur, M., Lassl, C., Lapin, A., and Haslberger, A.G. (2009). Combined PCR-DGGE fingerprinting and quantitative-PCR indicates shifts in fecal population sizes and diversity of Bacteroides, bifidobacteria and Clostridium cluster IV in institutionalized elderly. Exp. Gerontol. *44*, 440–446.

STAR★METHODS

KEY RESOURCES TABLE

REAGENT or RESOURCE	SOURCE	IDENTIFIER
Antibodies		
Anti-mouse F4/80-APC	eBioscience	Cat#17-4801-82; RRID: AB_469452
Anti-mouse Ly6G-PE	BD Biosciences	Cat#551461; RRID: AB_394208
Anti-mouse CD45-efluor450	eBioscience	Cat#48-0451; RRID: AB_1518806
Anti-mouse CD11b-PeCy7	BioLegend	Cat#301321; RRID: AB_830643
Anti-mouse TLR4-FITC	eBioscience	Cat#53-9041-82; RRID: AB_469944
Anti-mouse TLR2-PeCy7	eBioscience	Cat#25-9024-80; RRID: AB_469687
Anti-mouse CD14-PerCpCy5.5	eBioscience	Cat#45-0141; RRID: AB_925733
Anti-MARCO-PE	AbSerotec	Cat#0310
Anti-beta actin	Cell Signaling Technologies	Cat#4970; RRID: AB_2223172
Bacterial and Virus Strains		
Streptococcus pneumoniae strain P1547	Prof. Jeffrey Weiser	N/A
Biological Samples		
Mouse bone marrow derived or peritoneal macrophages	C57BL/6J or B6.129S-Tnftm1Gkl/J	Collected in house, age either 10-14 wk (young) or 18-22 mo (old)
Chemicals, Peptides, and Recombinant Proteins		
Adalimumab/Humira	Abbott Laboratories	N/A
Lidocaine powder	Sigma	Cat# L7757
Human IgG	BioLegend	Cat# 403102
Tryptic Soy Agar	BD	Cat# 211822
4kDa FITC-Dextran	Sigma	Cat#46944
Heparin Sodium Injection 1000 USP Units/mL	Sandoz	DIN 02303086
Chromium-51 Radionuclide, 1mCi, EDTA Complex in 0.005M EDTA	Perkin-Elmer	Cat#NEZ14700
Eschericia coli 055:B5, ultrapure	Invivogen	Cat# tlrl-pb5lps
Taq polymerase and buffer solution	Life Technologies	Cat# J00273
Critical Commercial Assays		
Mouse IL6 ELISA Ready-SET-Go	eBioscience	Cat# 88-7064
QiaQuick Gel Extraction	QIAGEN	Cat#28704
Milliplex Catalog ID.MCYTOMAG-70K-02.Mouse Cytokine MAGNETIC Kit	Milliplex	Cat#MCYTOMAG-70K-02
Deposited Data		
Microbiome data submitted	Bioproject ID: PRJNA379319	Bioproject ID: PRJNA379319
Experimental Models: Cell Lines		
HEK293T-NOD2/pNifty2-SEAP	Created in house	N/A
Experimental Models: Organisms/Strains		
Mouse: C57BL/6J 10-14 wk (young) or 18-22 mo (old), raised under either specific pathogen free or germ-free conditions	Jackson labs	000664
Mouse: B6.129S-Tnftm1Gkl/J 10-14 wk (young) or 18-22 mo (old)	Jackson labs	005540
Recombinant DNA		
Software and Algorithms		
Custom, in-house Perl scripts to process the sequences after Illumina sequencing	Whelan et al., 2014	Whelan et al. Ann Am Thorac Soc *11*, 513-521.
PANDAseq	Masella et al., 2012	BMC Bioinformatics *13*, 1-7.

(*Continued on next page*)

Continued		
REAGENT or RESOURCE	SOURCE	IDENTIFIER
Cutadapt	Martin, 2011	https://github.com/marcelm/cutadapt
Ribosomal Database Project (RDP) classifier	Michigan State University	https://rdp.cme.msu.edu/classifier/classifier.jsp
Greengenes reference database		http://greengenes.lbl.gov/cgi-bin/JD_Tutorial/nph-Alignment.cgi
AbundantOTU+	Indiana University	http://omics.informatics.indiana.edu/AbundantOTU/
Quantitative Insights into Microbial Ecology (QIIME)	Caporaso et al., 2010	Nat Methods *7*, 335-336.
Other		
Illumina 16 s rRNA sequencing	This paper	http://www.science.mcmaster.ca/mobixlab/

CONTACT FOR REAGENT AND RESOURCE SHARING

Further information and requests for resources and reagents should be directed to and will be fulfilled by the lead author, Dawn Bowdish (bowdish@mcmaster.ca).

EXPERIMENTAL MODEL AND SUBJECT DETAILS

Ethics statement

All experiments were performed in accordance with Institutional Animal Utilization protocols approved by McMaster University's Animal Research Ethics Board as per the recommendations of the Canadian Council for Animal Care.

Mouse Experiments

WT young (10-16 wk) and old (18-22 mo) C57BL/6 and $TNF^{-/-}$ mice (originally from Jackson Laboratories), were bred in house. To protect from age-related obesity, aging SPF mice (and corresponding young controls) are fed with a low protein diet Teklad Irradiated Global 14% protein Maintenance Diet and provided with an exercise wheel. The average weight of a young SPF mouse (8-14 wks) in this study is 20 g+/–1g and the old SPF mice (18-22 mo) are on average, 27 g+/–2.5g. Mice were housed in pathogen-free conditions and pathogen-free status of mice within the aging colony was confirmed in mice through constitutive monitoring of sentinel mice and specific testing of fecal samples for common mouse pathogens. Mice were maintained in the same animal room, with the exception of germ-free and ASF mice (all C57BL/6), which were bred in the Gnotobiotic Facility of McMaster. Because the WT and $TNF^{-/-}$ mice were originally from different breeding colonies from Jackson labs, and the ASF mice had fundamentally different microbiota, changes in the microbiota were made within strains (i.e., differences between young and old WT *or* young and old $TNF^{-/-}$ *or* young and old ASF mice) but not between strains. For experiments described in Figures 1, 2, 3, and 5 female mice were used. For experiments described in Figure 4 groups of sex matched male and female mice were used. For studies of the microbiota, in order to minimize cage effects or familial transfer of the microbiota (as described in (Ubeda et al., 2012)), mice were selected from multiple cages and multiple breeding pairs. No evidence of cage effects was found in any of the studies of the microbiota. Due to the limited availability of aged ASF mice, experiments were performed in groups of 2-4 mice from multiple breeders over 2 years, again minimizing cage effects.

METHOD DETAILS

TNF ablation

Adalimumab (HUMIRA, Abbott Laboratories), a humanized anti-TNF antibody, or the human IgG isotype control diluted in sterile saline were administered to mice. A dose of 40 μg per gram of body weight was given intraperitoneally in a volume of 200 μl every other day, for a period of 3 weeks to young and old WT mice.

Histological analysis

Histopathological analysis was carried out on samples from the lungs of old WT, TNF KO and germ-free mice, and their young controls. Upon collection, lungs were formalin-inflated and these, alongside formalin fixed spleens, were paraffin-embedded. Tissue blocks were cut into 5-μm sections that were stained with hematoxylin-eosin (HE). The slides were blinded/coded and the colon epithelial architecture and inflammation were histologically scored. Histological scoring was performed using the following system: tissue architectural changes: 0, normal; 1, blebbing; 2, loss of epithelium; 3, complete loss of crypt architecture; and inflammation: 0, normal; 1, increased number of inflammatory cells in lamina propria; 2, increased number of inflammatory cells in submucosa; 3, dense inflammatory cell mass, but not transmural in nature; 4, transmural inflammation. The average score for all mice was 0 for both inflammation and epithelial cell architectural changes. Cellular infiltration in the lungs was quantitated in H&E stained sections. The degree of inflammation within each lung was measured by expressing the total area of the cellular infiltrate within the lung as a

percentage of the total lung area using ImageJ. Images were acquired with a Leica DM LB2 microscope at a magnification of 20X and captured using a Leica DFC 280 camera.

Measurement of cytokine production

For circulating levels of cytokines, blood samples from naive animals were collected by retro-orbital bleeding into heparin, and spun at 1500 x *g* for 5 min. 100 μL of plasma was then collected, and IL6 levels assayed using ELISA as per the manufacturer's direction (eBioscience). To measure the TNF and IL6 cytokine concentrations within the plasma samples of the colonized germ-free mice, Milliplex immunoassay Kits were used and completed as recommended by the manufacturer's instructions (Millipore, Etobicoke, ON). For whole blood stimulation studies, 100 μL of whole blood samples collected in heparin from young and old WT, TNF KO and germ-free mice were stimulated with 100 ng/ml of LPS (*Eschericia coli* 055:B5, ultrapure Invivogen), or left unstimulated. Samples were incubated for 24 hr at 5% CO_2 and 37°C, then centrifuged at 1500 x *g* for 5 min. 50 μL of plasma samples were assayed for the presence of IL6 using ELISA. To measure constitutive levels IL6 in the lung, right lobe samples of lung were mechanically homogenized in 500 μL of PBS and assayed by ELISA. To measure inducible cytokine production in lung tissue, lungs were perfused with low melt agarose and sliced into 10 micron sections. 3 slices were cultured in 1 mL of media for 24 hr; supernatants were then removed and assayed for IL6 production using 100 μL of sample ELISA. To measure cytokine production by bone marrow macrophages, 3.5×10^5 mature bone-marrow-derived macrophages were seeded in a 24-well tissue culture-grade plate (Fisher) in 1.5 mL of media and allowed 24 hr to recover. Cells were then stimulated with either 100 ng/ml of LPS (*Eschericia coli* 055:B5, ultrapure Invivogen), whole heat-killed P1547 at an MOI of 50 or 50 μL of media control. Supernatants were collected at 24 hr post-stimulation. Levels of TNF or IL6 were measured by ELISA.

Macrophage culture

Bone marrow derived-macrophages were isolated according to previously published methods(*60*) and differentiated in the presence of L929 conditioned media for 8 days as per standard protocols. After 8 days the cells were incubated with 4 mg/ml lidocaine (Sigma) for 15 min at 4°C and gently lifted using a cell lifter. Cells were then centrifuged, counted and re-suspended in medium at a concentration appropriate for measurement of cytokine production, bacterial uptake, flow cytometry or bacterial killing assays. Macrophage maturation was assessed by flow cytometry using APC-conjugated anti-F4/80, PE-conjugated anti-Ly6G or -CCR2, FITC-conjugated Ly6C, eFluor 450-conjugated CD45 and PE-Cy7-conjugated CD11b, or corresponding isotype controls. Pattern recognition receptor (PRR) expression was measured using anti-TLR4-FITC, anti-TLR2-PE-Cy7 and anti-CD14-PerCpCy5.5 (eBioscience), as well as anti-MARCO-PE (RND systems). To visualize *S. pneumoniae* uptake by macrophages, TRITC labeled bacteria were incubated with bone marrow derived macrophages for 2h at an MOI of 200. Cells were fixed and stained using an anti-beta actin antibody (Cell Signaling). Images were acquired at 40X magnification using an inverted Zeiss LSM510 laser confocal microscope.

Bacterial killing assays

To measure macrophage killing of *S. pneumoniae,* 5×10^5 bone marrow derived macrophages were pre-incubated with an multiplicity of infection (MOI) of 10 bacteria per macrophage for 60 min at 37°C with gentle inversion as outlined above to allow for internalization of bacteria (Novakowski et al., 2017). Viable CFUs were determined by culturing of supernatants on TS agar plates.

In vitro and in vivo permeability

Sections of colon and ileum were excised, opened along the mesenteric border, and mounted in Ussing chambers (World Precision Instruments, Sarasota, Florida). Tissues (ileum and colon) were allowed to equilibrate for 15-25 min before baseline values for potential difference (PD) and short circuit current (Isc) were recorded. Tissue conductance (G) was calculated by Ohm's law using the PD and Isc values. Mucosal to serosal flux of the small inert probe (360 Da) ^{51}*chromium*-ethylenediaminetetraacetic acid (^{51}Cr-EDTA) was used to assess paracellular permeability. After equilibration, time zero samples were taken from the serosal buffer and 6μCi/ml ^{51}CR-EDTA was added to the mucosal compartment. A "hot sample" was taken from the mucosal buffer then samples were then taken every 30 min from the serosal buffer for 2 hr and counted in a liquid scintillation counter (Beckman). Counts from each 30 min were averaged and compared to the "hot sample"(100%). Data expressed as mucosal-to-serosal flux (%flux/cm^2/hr). Each sample was completed in duplicates. Recordings were performed as described previously (Slack et al., 2009; Verdu et al., 2008).

For non-terminal studies, tracer FITC-labeled dextran (4kDa; Sigma-Aldrich) was used to assess in vivo intestinal permeability. Mice were deprived of food 4 hr prior to and both food and water 4 hr following an oral gavage using 200 μl of 0.8 mg/ml FITC-dextran. Blood was retro-orbitally collected after 4 hr, and fluorescence intensity was measured on fluorescence plates using an excitation wavelength of 493nm and an emission wavelength of 518 nm.

MDP Detection Bioassay

HEK293T cells stably were transfected with mNod2 (a kind gift from Dr. Jonathan Schertzer) and pNifty2-SEAP plasmids (Invivogen) to create a reporter system. Binding of the intracellular mNod2 receptor with its ligand, MDP, results in downstream activation and translocation of NFκB. Activation of this transcription factor leads to SEAP expression via the ELAM proximal promoter, which is detected via absorbance spectroscopy. Plates were seeded with cells 24 hr prior to addition of heat-inactivated mouse plasma, diluted

1 in 200 in HEK Blue Detection Media (Invivogen) to a final volume of 200 μl, in a 96-well plate format. Readings were performed at 630nm, 24 hr subsequent to stimulation as described in (Verschoor et al., 2015).

Germ-free Mouse Recolonization

For recolonization studies, one young and old germ-free mice were transferred to individually ventilated racks and co-housed with either a young or old mouse. Due to the availability of aged germ free mice, 8 independent colonization experiments of 2-6 young or old germ-free mice were performed over 3.5 years. Consequently the SPF mice that were used were from different breeding pairs, ensuring that cage effects or changes specific to a particular breeding pair were minimized. The mice were left undisturbed for two week following the start of the colonization and then maintained for a minimum of 6 weeks at which point fecal pellets were collected for microbiome analysis (as described below), plasma cytokines were assayed and intestinal permeability was measured as described above.

Bacterial profiling by deep sequencing analysis of 16S rRNA with Illumina

Fecal pellets were collected and the V3 region of the 16S rRNA gene was amplified by PCR as in Bartram et al. (2011); Stearns et al. (2015); and Whelan et al. (2014). Briefly, each 50 μL PCR reaction mixture contained 1.5 mM of $MgCl_2$ (50mM), 200 μM dNTPs, 4 mM of BSA, 25 pmol of each primer, 1U of Taq polymerase (Life Technologies), and 200 ng of DNA. The reaction was then run for 30 cycles (94°C for 2 min, 94°C for 30 s, 50°C for 30°C, 72°C for 30 s), with a final polymerization step at 72°C for 10 min (Eppendorf). The products were separated by electrophoresis in 2% agarose gel and visualized under a UV transilluminator and the products corresponding to the amplified V3 region (~300 base pairs) were excised and purified using standard gel extraction kits (QIAGEN). Illumina sequencing and initial quality control were carried out by the MOBIX-McMaster Genome Center (McMaster University). Custom, in-house Perl scripts were developed to process the sequences after Illumina sequencing (Whelan et al., 2014). Briefly, Cutadapt was used to trim the forward and reverse paired-end reads at the opposing primers for input into PANDAseq for assembly (Martin, 2011; Masella et al., 2012). Mismatches and ambiguous base attributions in the assembly from specific set of paired end sequences were discarded. Operational taxonomic units (OTUs) were picked using AbundantOTU+ and taxonomy-assigned using the Ribosomal Database Project (RDP) classifier against the Greengenes reference database (Ye, 2011). OTU number are generated in order from most abundant (OTU 1) when clustering using AbundantOTU +. Single sequence OTUs (singletons) were removed prior to all analyses using Quantitative Insights into Microbial Ecology (QIIME) (Caporaso et al., 2010).

QUANTIFICATION AND STATISTICAL ANALYSIS

Unless otherwise mentioned in the figure legend, statistical significance was determined by two-way analysis of variance with Fischer's post-test and unpaired t tests (two tailed). Statistical significance was defined as a *p* value of 0.05. All data were analyzed with Prism (Version 6; GraphPad). Differences in the survival curves were analyzed by Log-rank (Mantel-Cox) test Microbiota changes were analyzed with Quantitative Insights into Microbial Ecology (QIIME) software using principal component analysis as measured by Bray-Curtis. The Chi-square of the likelihood ratio test in phyloseq DESeq2 was used to determine differences between groups as in (McMurdie and Holmes, 2013). In order to avoid the challenges of multiple testing correction, two datasets for the young and old microbiota samples were generated from samples gathered approximately 6 months apart from at least 5 different cages of mice. A list of OTUs representing families or genuses which changed in abundance in old SPF mice was created and statistically significant differences in the second dataset were determined using Welch's unequal variances t test. Data from the second dataset are presented in Tables 1 and 2. No evidence of cage effects was found. Bray-Curtis distances were calculated and interactions between age and treatment were tested using the permanova test 'adonis' from the 'vegan' package in R.

DATA AND SOFTWARE AVAILABILITY

All data are available upon request to the lead contact author. No proprietary software was used in the data analysis. The accession number for the data reported in this paper is Bioproject ID: PRJNA379319.

Cell Host & Microbe, Volume *21*

Supplemental Information

Age-Associated Microbial Dysbiosis Promotes Intestinal Permeability, Systemic Inflammation, and Macrophage Dysfunction

Netusha Thevaranjan, Alicja Puchta, Christian Schulz, Avee Naidoo, J.C. Szamosi, Chris P. Verschoor, Dessi Loukov, Louis P. Schenck, Jennifer Jury, Kevin P. Foley, Jonathan D. Schertzer, Maggie J. Larché, Donald J. Davidson, Elena F. Verdú, Michael G. Surette, and Dawn M.E. Bowdish

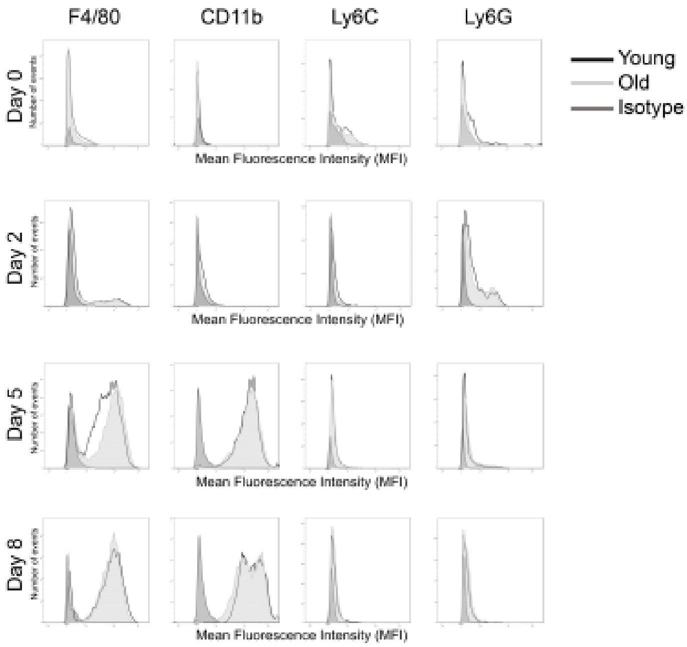

Figure S1 related to Figure1. Macrophage maturation does not differ with age. Histograms showing expression of key maturity markers (CD11b, F4/80, Ly6C and Ly6G) on differentiating bone marrow at days 0, 2, 5 and 8. Bone marrow was derived from young (3 mo and old (18-20 mo) mice and differentiated in M-CSF-enriched media. Bone marrow from one representative mouse of at least three is shown; cells were analyzed using flow cytometry. There were no statistically significant differences in the levels of expression of the markers tested at Day 8, when macrophages were used for experiments.
